# Prediction of post-radiotherapy survival for bone metastases: a comparison of the 3-variable number of risk factors model with the new Katagiri scoring system

**DOI:** 10.1093/jrr/rrab121

**Published:** 2021-12-31

**Authors:** Takayuki Sakurai, Shigeyuki Takamatsu, Nana Shimoyachi, Satoshi Shibata, Mikoto Makino, Shizuko Ohashi, Yoko Taima, Risako Minamikawa, Tomoyasu Kumano, Toshifumi Gabata

**Affiliations:** Department of Radiology, Kanazawa University Graduate School of Medical Sciences, Kanazawa, Ishikawa, Japan; Department of Radiology, Kanazawa University Graduate School of Medical Sciences, Kanazawa, Ishikawa, Japan; Department of Radiation Oncology, Cancer Institute Hospital of the Japanese Foundation for Cancer Research, Tokyo, Japan; Department of Radiology, Kanazawa University Graduate School of Medical Sciences, Kanazawa, Ishikawa, Japan; Department of Therapeutic Radiology, Kanazawa Medical Center, Kanazawa, Ishikawa, Japan; Radiation Therapy Center, Fukui Saiseikai Hospital, Fukui, Japan; Department of Therapeutic Radiology, Ishikawa Prefectural Central Hospital, Kanazawa, Ishikawa, Japan; Department of Radiology, Kanazawa University Graduate School of Medical Sciences, Kanazawa, Ishikawa, Japan; Department of Radiology, Graduate School of Medicine, Gifu University, Gifu, Japan; Department of Radiology, Kanazawa University Graduate School of Medical Sciences, Kanazawa, Ishikawa, Japan

**Keywords:** bone metastasis, new Katagiri scoring system, 3-variable number of risk factors (NRF) model, palliative radiation therapy, prognostic factors

## Abstract

We investigated patient survival after palliative radiotherapy for bone metastases while comparing the prognostic accuracies of the 3-variable number of risk factors (NRF) model and the new Katagiri scoring system (Katagiri score). Overall, 485 patients who received radiotherapy for bone metastases were grouped as per the NRF model (groups I, II and III) and Katagiri score (low-, intermediate- and high-risk). Survival was compared using the log-rank or log-rank trend test. Independent prognostic factors were identified using multivariate Cox regression analyses (MCRA). MCRA and receiver operating characteristic (ROC) curves were used to compare both models’ accuracy. For the 376 evaluable patients, the overall survival (OS) rates decreased significantly in the higher-tier groups of both models (P < 0.001). All evaluated factors except ‘previous chemotherapy status’ differed significantly between groups. Both models exhibited independent predictive power (P < 0.001). Per NRF model, hazard ratios (HRs) were 1.44 (P = 0.099) and 2.944 (P < 0.001), respectively, for groups II and III, relative to group I. Per Katagiri score, HRs for intermediate- and high-risk groups were 4.02 (P < 0.001) and 7.09 (P < 0.001), respectively, relative to the low-risk group. Areas under the curve (AUC) for predicting 6-, 18- and 24-month mortality were significantly higher when using the Katagiri score (P = 0.036, 0.039 and 0.022). Both models predict survival. Prognostic accuracy of the Katagiri score is superior, especially in patients with long-term survival potential; however, in patients with short prognosis, no difference occurred between both models; simplicity and patient burden should also be considered.

## INTRODUCTION

Bone metastases are a common manifestation of malignant tumors that can cause pain and spinal cord compression [1]. Radiotherapy provides excellent palliation for painful bone metastases [1–3]. According to the American Society for Radiation Oncology (ASTRO) evidence-based guidelines, commonly used radiotherapy dose fractionation schedules include 30 Gy (3 Gy/fraction), 24 Gy or 20 Gy (4 Gy/fraction) and 8 Gy (single fraction); all such schedules reportedly result in notable pain relief [1], and single-fraction radiotherapy has the advantage of being more convenient for patients and caregivers [1]. Moreover, there exists high-quality evidence supporting single-fraction radiotherapy for patients with a limited life expectancy, and it is important for the radiation oncologist to determine the dose fractionation, taking into account various factors such as the patient’s general condition and life expectancy [2]. In this respect, life expectancies predicted by clinicians have often been reported to be inaccurate [4–6].

In 2003, Chow *et al.* published a prognostic model for patients with metastatic cancer receiving palliative radiotherapy [7], whereas in 2008, they reported the simpler 3-variable number of risk factors (NRF) model that utilizes only three predictive factors [8]. Several studies have validated the NRF model and found it to be useful [9–11]. Subsequently, Katagiri *et al.* reported a prognostic model for patients with bone metastases who underwent surgery or radiotherapy for bone metastases in 2005 [12], and published the new Katagiri scoring system (Katagiri score) with additional modifications in 2014 [13]. Several investigators have utilized Katagiri *et al.*’s prognostic model when determining the radiotherapy schedule [14–18], including in a validation study [17]. Although other investigators have reported prognostic models that include only patients who underwent radiotherapy for spinal metastases [19, 20], both the NRF model and Katagiri score can predict the prognoses not only of patients with spinal metastases but also of those with bone metastasis at any site, and are also suitable for patients who undergo palliative radiotherapy. However, it remains unclear which of these two models is more accurate given the lack of studies comparing them.

In this study, we retrospectively investigated the survival intervals of patients who underwent palliative radiotherapy for bone metastases. Additionally, we evaluated every patient according to the NRF and Katagiri score methods and compared the methods’ accuracies.

## MATERIALS AND METHODS

### Patients

This study was approved by the institutional review board of our hospital (no. 2017-001[2438]). Between April 2011 and May 2020, 485 patients underwent radiotherapy for bone metastases; however, those who had previously received radiotherapy for bone metastases were excluded from the study. We retrieved the patients’ demographic information, primary cancer site data, survival data, medical image information, laboratory data, treatment history and general condition from medical records in 2021, taking ethical considerations into account. At the first visit to the radiotherapy department, the patients’ prognoses were assessed using the Katagiri score and the NRF model.

### The 3-variable number of risk factors model

The patients were grouped according to the total NRF: (i) non-breast cancer, (ii) sites of metastases other than bone, and (iii) a Karnofsky performance scale (KPS) score ≤ 60. Patients with one or no risk factors were classified as group I, those with two risk factors were assigned to group II, and those with three risk factors were classified as group III [8].

### The new Katagiri scoring system

To group patients according to the Katagiri score, 3 points were assigned to patients with rapid growth of the primary site tumor and 2 points for those with moderate growth. Disseminated metastasis was assigned 2 points while ordinary metastasis was allocated 1 point. In terms of laboratory data, 2 points were assigned for critical data and 1 point for abnormal data. Poor performance status (PS) as defined by the Eastern Cooperative Oncology Group (ECOG) score, previous chemotherapy and multiple bone metastases were each worth 1 point (Supplementary Table 1) [13]. Patients with total scores of 0–3 were categorized into the low-risk group, those with scores of 4–6 the intermediate-risk group, and those with scores of 7–10 the high-risk group.

### Radiotherapy

Radiotherapy was performed using a linear accelerator with 4, 6 or 10 MV photons; 3-dimensional treatment planning systems were implemented for all patients. The radiotherapy schedule was determined by the radiation oncologist taking into account the purpose of the treatment (such as pain relief and improvement of spinal cord compression) as well as the patient’s general condition, prognosis and presence of oligometastasis.

### Statistical analyses

The intervals were calculated from the date of the start of radiotherapy to that of event occurrence. Overall survival (OS) was estimated using the Kaplan–Meier method, and the survival curves of two groups were compared using the log-rank test while the trend of survival curves between three groups was tested using the log-rank trend test. Furthermore, multivariate analyses using Cox proportional hazards regression were used to identify the most significant predictors and prognostic models of OS. Receiver operating characteristic (ROC) curves were constructed and areas under the curve (AUCs) were calculated to evaluate the models’ performance in predicting mortality at 3, 6, 12, 18 and 24 months. If the follow-up period of any surviving or dropped-out patients was shorter than the respective time points in the 3-month, 6-month, 12-month, 18-month and 24-month ROC evaluations, those patients were excluded. Statistical significance was defined as P < 0.05. All statistical analyses were performed using EZR (Saitama Medical Center, Jichi Medical University, Saitama, Japan) [21].

**Table 1:** Patient characteristics (n = 376)

					value	(% or range)
Age (years)					67	(3–88)
Sex, n (%)						
Male					236	(62.8)
Female					140	(37.2)
Follow-up period (months)					5.8	(0–68)
Primary site, n (%)						
Lung					102	(27.1)
Liver					48	(12.8)
Gastrointestinal					37	(9.8)
Prostate					29	(7.7)
Breast					26	(6.9)
Others					134	(33.0)
Laboratory data, n (%)						
Normal					63	(16.8)
Abnormal					250	(66.4)
Critical					63	(16.8)
Visceral metastases, n (%)						
No					101	(26.9)
Nodular metastasis					181	(48.1)
Disseminated metastasis					94	(25)
PS, n (%)						
ECOG		0	KPS	100-90	42	(11.2)
ECOG		1	KPS	80-70	130	(34.6)
ECOG		2	KPS	60-50	99	(26.3)
ECOG		3	KPS	40-30	98	(26.1)
ECOG		4	KPS	20-10	7	(1.9)
Previous chemotherapy, n (%)						
No					85	(22.6)
Yes					291	(77.4)
Multiple bone metastases, n (%)						
No					89	(23.7)
Yes					287	(76.3)
The NRF model, n (%)						
Group I					63	(16.8)
Group II					163	(43.4)
Group III					150	(39.9)
The Katagiri scoring system, n (%)						
Low-risk group					33	(8.8)
Intermediate-risk group					159	(42.3)
High-risk group					184	(48.9)

Values are presented as median (range) unless otherwise noted.

PS = performance status, ECOG = Eastern Cooperative Oncology Group, KPS = Karnofsky performance score, NRF = number of risk factors

## RESULTS

### Patients’ clinicopathological characteristics

Of the 485 patients, 109 could not be evaluated using the Katagiri score owing to the lack of laboratory data and were therefore excluded. The characteristics of the remaining 376 patients who were included in the analysis are shown in Table 1. The median follow-up duration was 5.8 months (range: 0.0–68.0 months). The median follow-up duration of the surviving patients was 9.4 months (range: 0.2–68.0 months). The most common primary sites were the lung (27.1%), liver (12.8%), gastrointestinal tract (9.8%), prostate (7.7%) and breast (6.9%). The 376 patients analyzed included five pediatric patients (four with neuroblastoma and one with osteosarcoma) and 11 adolescent and young adult patients (four with gastrointestinal tract cancer, two with cancer of unknown primary, and five with other cancers). According to the NRF model, a plurality of patients (163, 43.4%) were classified as group II with a moderate prognosis; according to the Katagiri score, a plurality (184, 48.9%) were classified as high-risk indicating the poorest prognosis. The 109 excluded patients were classifiable using the NRF model, with 29, 42 and 38 patients in groups I, II and III, respectively (Supplementary Table 2).

### Radiotherapy

The total radiotherapy dose ranged from 8 to 66 Gy. Seventy-three patients (19.4%) received radiotherapy at 20 Gy in 5 fractions, 62 (16.5%) at 30 Gy in 10 fractions, 38 (10.1%) at 39 Gy in 13 fractions, 28 (7.4%) at 50 Gy in 25 fractions, 26 (6.9%) at 40 Gy in 20 fractions, 21 (5.6%) at 8 Gy in one fraction and 128 were administered as other fractionation plans.

### Overall survival

**Fig. 1.**
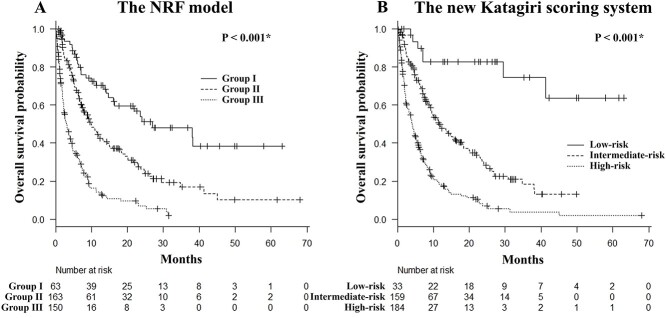
Kaplan–Meier curves of OS. Kaplan–Meier curves plotted to estimate the OS of patients grouped according to the 3-variable NRF model (A) and the new Katagiri scoring system (B). NRF = number of risk factors. ^*^Significant difference per the log-rank trend test (P < 0.05).

The Kaplan–Meier curve of OS is shown in Supplementary Fig. 1; the median survival time was 7.3 months (95% confidence interval [CI]: 6.4–9.1 months). The OS rates as determined according to the NRF model and Katagiri score are shown in Fig. 1 (Fig. 1A and 1B, respectively). In both prognostic models, the OS rate decreased significantly from groups I to III as well as from the low-risk to the high-risk groups (log-rank trend tests, P < 0.001). Using the NRF model, the median survival times of groups I, II and III were 27.0 months (95% CI: 14.7– NE [not estimable] months), 9.9 months (95% CI: 7.6–13.2 months) and 3.0 months (95% CI: 2.2–4.1 months). Per the Katagiri score, the low-risk group had an OS rate greater than 50%, and the median survival time had not been reached (95% CI: 29.5–NE months). The median survival time of the intermediate- and the high-risk groups were 11.8 months (95% CI: 9.1–16.1 months) and 4.1 months (95% CI: 2.8–5.2 months), respectively. In the excluded group of 109 patients who could only be evaluated using the NRF model, the OS rate decreased significantly from group I to group III, as in the group of 376 patients (log-rank trend test, P < 0.001; Supplementary Fig. 2).

### Multivariate analyses of prognostic factors according to each prediction model

Table 2 shows the results of the multivariate analyses using Cox regression to identify the prognostic factors most relevant to each prediction method. When using the NRF model, having metastatic sites other than the bone was most strongly associated with prognosis (P < 0.001, hazard ratio [HR] 2.94, 95% CI: 2.10–4.12). Using the Katagiri score, PS was the factor most associated with prognosis (P < 0.001, HR 3.22, 95% CI: 2.42–4.29). Having undergone previous chemotherapy was significantly associated with survival in univariate analysis (P < 0.001, HR 2.012, 95% CI: 1.43–2.83) but not in multivariate analyses (P = 0.429, HR 1.16, 95% CI: 0.81–1.67).

**Table 2:** Evaluation of prognostic factors for each scoring system by multivariate Cox regression analysis

The NRF model			The new Katagiri scoring system		
Factor	P value	HR (95% CI)	Factor	P value	HR (95% CI)
Non-breast cancer	0.002^^*^^	2.07 (1.24-3.47)	Primary-site-related factor	< 0.001^^*^^	1.53 (1.30-1.80)
Metastases other than bone	< 0.001^^*^^	2.94 (2.10-4.12)	Laboratory data	0.004^^*^^	1.39 (1.11-1.74)
KPS	< 0.001^^*^^	2.17 (1.68-2.81)	Visceral metastases	< 0.001^^*^^	1.71 (1.42-2.07)
			ECOG PS	< 0.001^^*^^	3.22 (2.42-4.29)
			Previous chemotherapy	0.425	1.16 (0.81-1.67)
			Multiple skeletal metastases	0.002^^*^^	1.58 (1.15-2.16)

NRF = number of risk factors, HR = hazard ratio, CI = confidence interval, ECOG = Eastern Cooperative Oncology Group, PS = performance status, KPS = Karnofsky performance score

^^*^^Significant difference by cox regression model (P < 0.05)

### Comparison of overall survival when using the number of risk factors model versus the Katagiri score

**Fig. 2.**
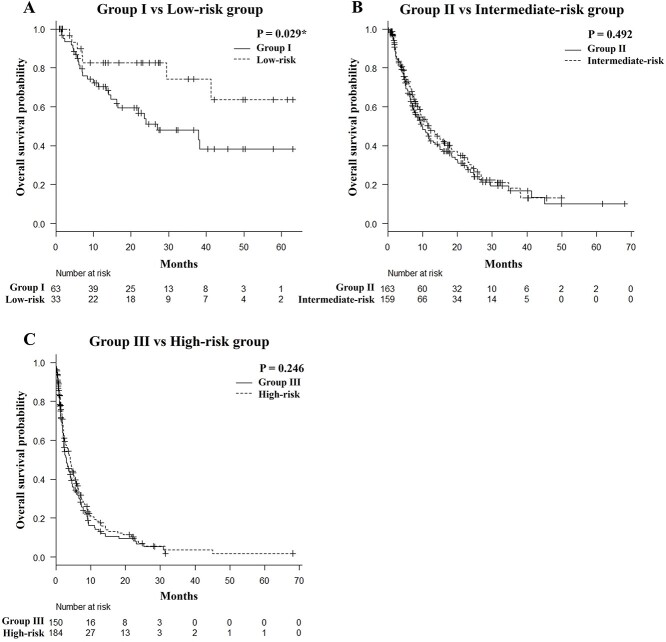
Comparison of OS: NRF model versus the Katagiri score. Comparison of survival curves between group I and the low-risk group (A), group II and the intermediate-risk group (B), and group III and the high-risk group (C). ^*^Significant difference using the log-rank test (P < 0.05).

Fig. 2 shows the comparison of survival curves between group I (NRF) and the low-risk group (Katagiri score) (Fig. 2A), group II (NRF) and the intermediate-risk group (Katagiri score) (Fig. 2B) and group III (NRF) and the high-risk group (Katagiri score) (Fig. 2C). The low-risk group (Katagiri score) achieved significantly longer survival than group I (NRF) (P = 0.029). There was no significant difference between group II (NRF) and the intermediate-risk group (Katagiri score), nor between group III (NRF) and the high-risk group (Katagiri score) (P = 0.492 and P = 0.246, respectively); the survival curves were almost identical.

In multivariate analyses using each prognostic model as a variable (Table 3), both models were independent predictors of OS (both *P* < 0.001); the HR of the Katagiri score (2.02) was higher than that of the NRF model (1.82). In multivariate analyses using each group as a variable (Table 3), the HRs with group I as the reference were 1.44 (*P* = 0.099, 95% CI: 0.93–2.23) and 2.94 (P < 0.001, 95% CI: 1.85–4.68) for groups II and III, respectively. In contrast, when using the low-risk group as the reference, the HRs were 4.02 (*P* < 0.001, 95% CI: 1.84–8.78) and 7.09 (P < 0.001, 95% CI: 3.19–15.78) for the intermediate- and high-risk groups, respectively.

**Fig. 3.**
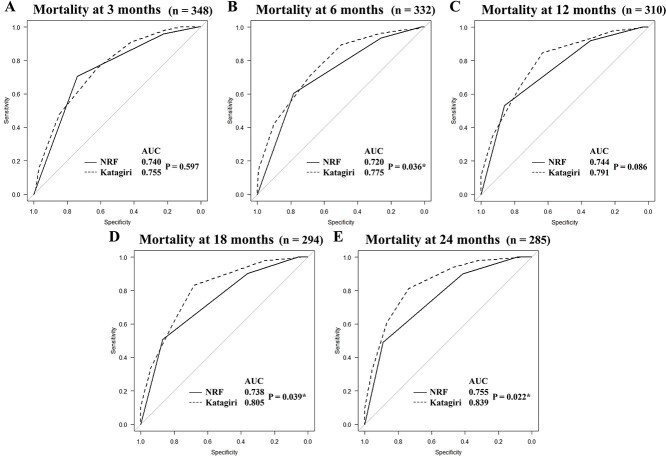
ROC curves for predicting mortality. ROC curves plotted for predicting mortality at 3 months (A), 6 months (B), 12 months (C), 18 months (D), and 24 months (E), using each prognostic model. NRF = number of risk factors model, Katagiri = new Katagiri scoring system, AUC = area under the curve. The total score in each prognostic model has been used as an explanatory variable ^*^Significant difference (P < 0.05).

The AUCs of the ROCs for predicting mortality at 3, 6, 12, 18 and 24 months using each prognostic model are shown in Fig. 3A–E (the total score is the explanatory variable). The AUCs of the Katagiri score for predicting mortality at 6, 18 and 24 months were significantly higher than those obtained using the NRF model (P = 0.036, P = 0.039 and P = 0.022, respectively).

Based on these findings, the risk classification according to the Katagiri score was deemed more accurate than the risk classification according to the NRF model in terms of predicting the OS of patients undergoing radiotherapy for bone metastases, especially in patients with long-term survival potential.

## DISCUSSION

We found that the NRF and Katagiri scores are both independent predictors of OS in patients receiving radiotherapy for bone metastases. Moreover, all factors comprising each prognostic model (except the Katagiri variable ‘previous chemotherapy’) were independent predictors of prognosis. The Katagiri score was superior to the NRF model in terms of its prognostic accuracy, especially in patients with long-term survival potential. However, no difference in survival was observed between both models in the group of patients with a shorter prognosis (groups II and III in the NRF model). To our knowledge, ours is the first study to compare the accuracy of the NRF model to that of the Katagiri score in patients undergoing radiotherapy for bone metastases.

Although the prognostic accuracy of the Katagiri score was superior to that of the NRF model, the NRF model has the advantage of being very simple and not requiring invasive blood sampling or detailed imaging studies as are necessary for assessing the Katagiri score. Moreover, some physicians may hesitate to perform invasive tests to estimate a patient’s prognosis if there are no recent laboratory data at the time palliative radiotherapy is administered. Westhoff *et al.* reported a very simple prognostic tool that combines the KPS and primary site only (not a scoring system) and showed comparable prediction accuracy to that of the most complex model they developed [22]. Prognostic models for palliative radiotherapy are not frequently used in practice [23], perhaps because of their complexity. We believe that accuracy is the most important factor in selecting a prognostic model for clinical use from among the many models, but we also believe that simplicity is one of the important factors. In the current study, 109 patients were excluded because the Katagiri score could not be assessed; thus, predicting prognosis using the NRF model would benefit more patients. The prognoses of patients in the Katagiri score low-risk group were better than those in the NRF model group I (Fig. 2A), with the Katagiri score AUCs increasing from 3 months (AUC 0.755) to 6 months (AUC 0.775) to 12 months (AUC 0.791) to 18 months (AUC 0.805) and finally to 24 months (AUC 0.839) (Fig. 3). As such, the Katagiri score was able to identify patients who were likely to achieve long-term survival. Taken together, we suggest first assessing patients using the simpler NRF model; if the patient is found to be classified into group III, we suggest performing an 8 Gy single fraction. On the other hand, if a patient is found to be in group I, then assessment using the Katagiri score can be considered. If a patient is in the Katagiri score low-risk group, we suggest considering a longer radiotherapy course; stereotactic body radiotherapy may also be used in case of oligometastatic disease.

**Table 3:** Multivariate analysis with cox regression to compare the accuracy of prognostic models

Variables (Covariables)	P value	HR (95% CI)	
Multivariate analysis with each prognostic model as a variable			
Prognostic model			
The NRF model	< 0.001^^*^^	1.82 (1.47-2.26)	
The new Katagiri scoring system	< 0.001^^*^^	2.02 (1.59-2.57)	
Multivariate analysis with each group as a variable			
The NRF model			
Group I	-	1	-
Group II	0.099	1.44 (0.93-2.23)	
Group III	< 0.001^^*^^	2.94 (1.85-4.68)	
The new Katagiri scoring system			
Low-risk group	-	1	-
Intermediate-risk group	< 0.001^^*^^	4.02 (1.84-8.78)	
High-risk group	< 0.001^^*^^	7.09 (3.19-15.78)	

NRF = number of risk factors, HR = hazard ratio, CI = confidence interval

^^*^^Significant difference by cox regression model (P < 0.05)

Regarding the survival of patients undergoing palliative radiotherapy, studies of prognostic models focusing only on spinal metastases or spinal cord compression have found that the primary tumor growth, visceral metastasis, ECOG PS and KPS were critical; this is similar to the Katagiri score and NRF model [19, 20]. In our study, ECOG PS showed the strongest association with survival per the Katagiri score. Many studies have shown that the KPS or ECOG PS is significantly correlated with survival [4, 8, 20, 22, 24–27]. As such, the continuous assessment of the KPS and ECOG PS is warranted in daily practice. Katagiri *et al.* found that previous chemotherapy was a significant prognostic factor [13], but was the sole non-significant factor per our multivariate analyses (Table 2). A few studies found that treatment with molecularly targeted drugs is an important predictor of prognosis in patients with bone metastases arising from lung cancer [28, 29]. The Katagiri score variable ‘primary tumor growth’ also evaluates whether lung cancer can be treated with molecularly targeted drugs. However, such drugs have also been approved for many other types of cancers in the past few years, and more recently, several immune checkpoint inhibitors have been approved for various cancers, causing a paradigm shift in cancer drug therapy [30]. Gregory *et al.* and the Japanese Society of Medical Oncology guidelines for the treatment of bone metastases suggested a reconsideration of the prognostic models in the era of molecularly targeted drugs [31, 32]. As such, the use of these agents may have influenced the applicability of the ‘previous chemotherapy’ factor. In the future, it may be necessary to exclude factors such as ‘previous chemotherapy,’ for which no significant difference was observed, ‘laboratory data,’ which had many missing data, and to add factors such as ‘molecularly targeted drugs’ and ‘immune checkpoint inhibitors’ to construct an optimal prognostic model.

This study has limitations inherent to retrospective studies, such as selection and information biases. As a single-center study, it has further limitations. In addition, due to the lack of laboratory data, some patients were excluded from the analysis because the Katagiri score could not be assessed, which may have caused sampling bias. Further multicenter prospective studies are needed to replicate and confirm the findings of this study.

In conclusion, the NRF model and Katagiri score are both independent predictors of OS in patients receiving radiotherapy for bone metastases. In terms of prognostic accuracy, the Katagiri score is superior to that of the NRF model, especially in patients who were likely to achieve long-term survival; however, the NRF model remains far simpler to use. We recommend a single fraction if the patient is in group III on the NRF model, or a longer course if the patient is in the low-risk group in the Katagiri score. The most appropriate model for each patient ought to be selected while taking into account the burden on the individual and whether the laboratory and imaging data can be evaluated.

## Supplementary Material

Supplementary_Figure_1_rrab121

Supplementary_Figure_2_rrab121

Supplementary_Table_1_rrab121

Supplementary_Table_2_rrab121

Supplementary_rrab121

## Data Availability

Research data are stored in an institutional repository and will be shared upon request to the corresponding author.

## PRESENTATION AT A CONFERENCE

Shimoyachi N *et al*.: Evaluation of prognostic factors of bone metastases treated by radiotherapy: a single-institution experience. RSNA 104th Scientific Assembly and Annual Meeting, 2018.
