# Supplementary figures and images for: Prediction of post-radiotherapy survival for bone metastases: a comparison of the 3-variable number of risk factors model with the new Katagiri scoring system
Source: J Radiat Res. 2021 Dec 31;63(2):303–11. doi: 10.1093/jrr/rrab121 (PMC8944300; doi:10.1093/jrr/rrab121)

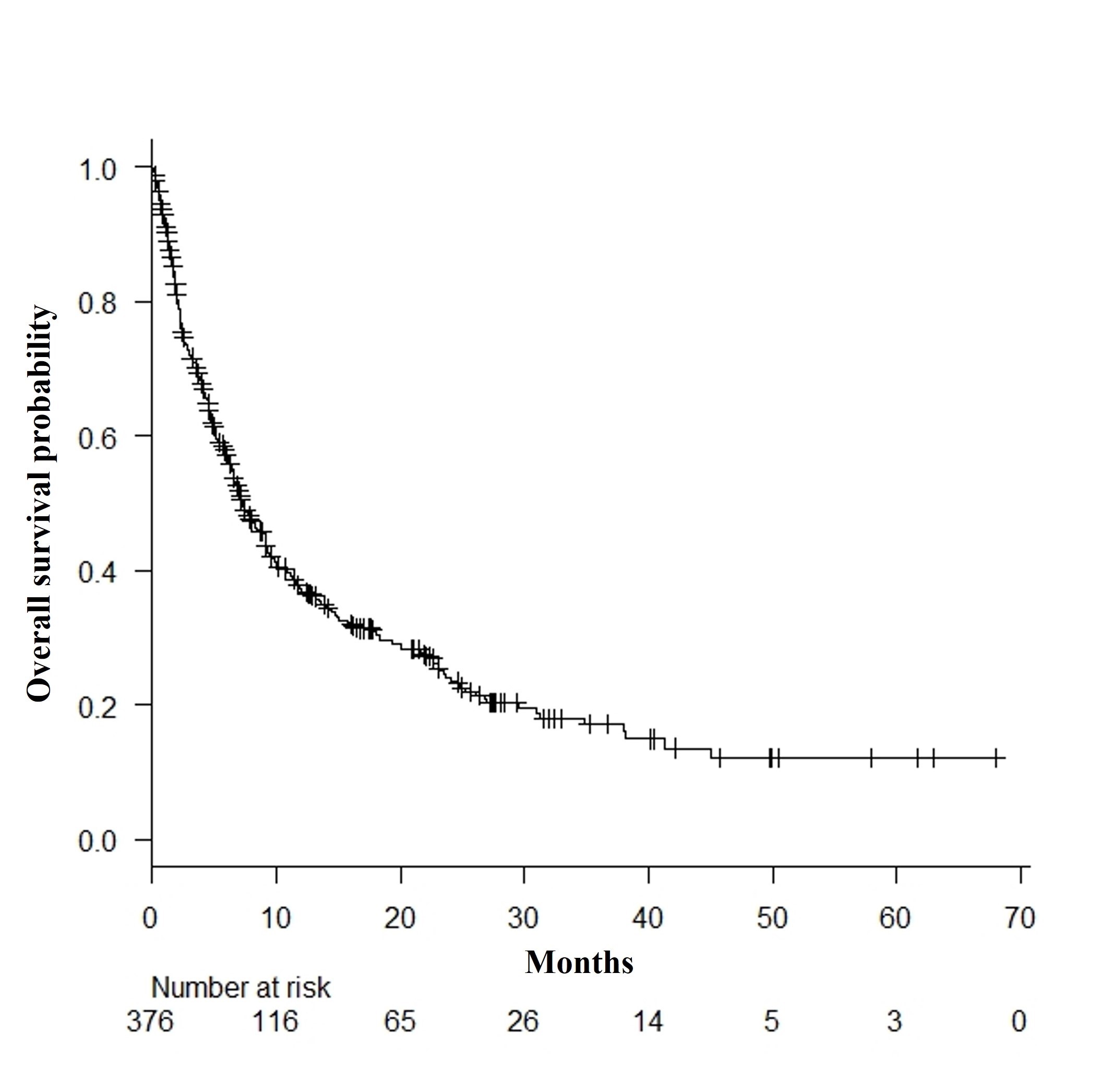

Supplement: Supplementary_Figure_1_rrab121 [file supplementary_figure_1_rrab121.jpeg]

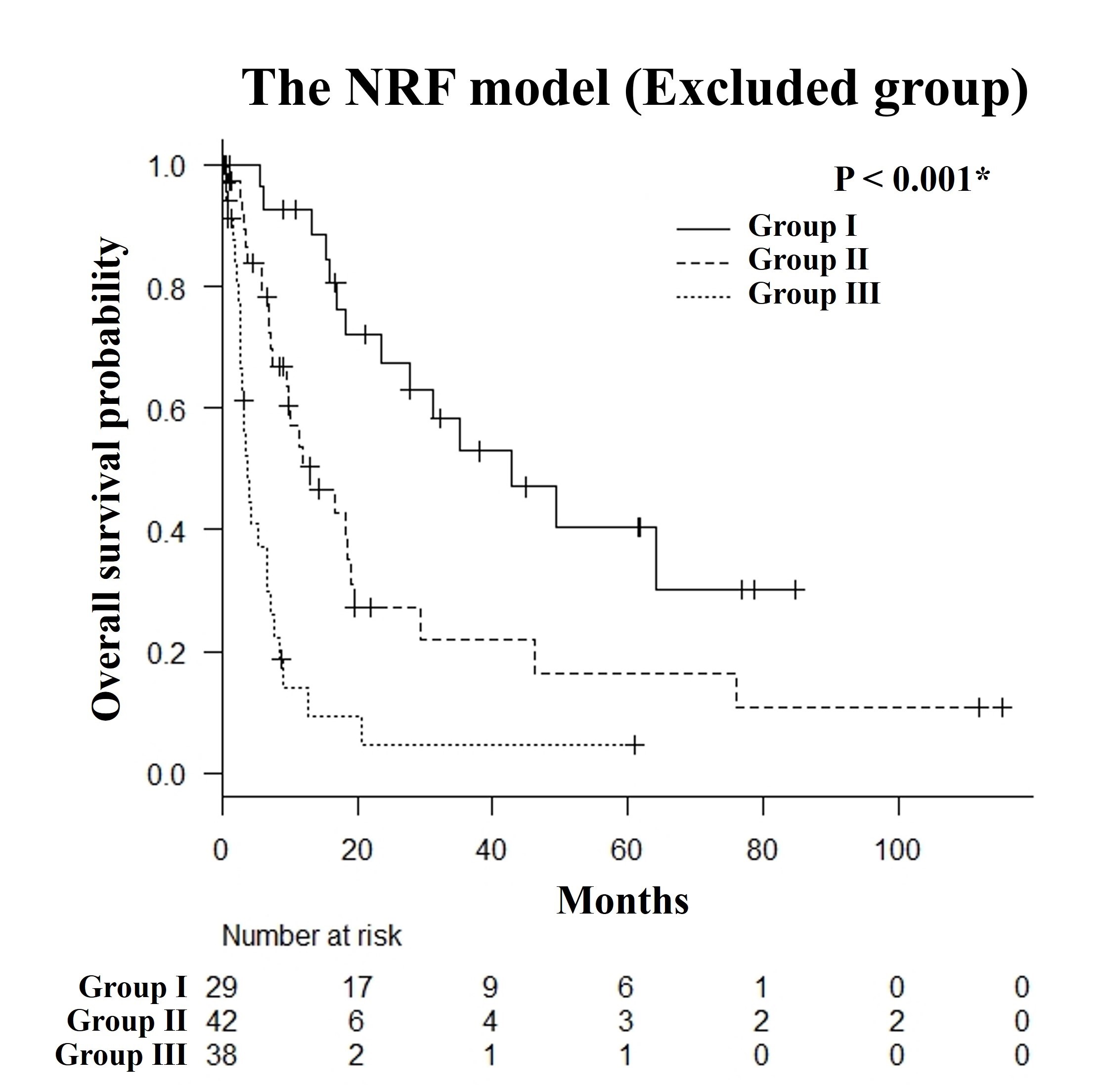

Supplement: Supplementary_Figure_2_rrab121 [file supplementary_figure_2_rrab121.jpeg]
