# Supplementary material for: Prediction of post-radiotherapy survival for bone metastases: a comparison of the 3-variable number of risk factors model with the new Katagiri scoring system
Source: J Radiat Res. 2021 Dec 31;63(2):303–11. doi: 10.1093/jrr/rrab121 (PMC8944300; doi:10.1093/jrr/rrab121)
Supplement: Supplementary_Table_1_rrab121 [file supplementary_table_1_rrab121.docx]

**Supplementary Table 1.** **The new Katagiri scoring system**

| **Prognostic factor** |  |  | **Score** | |  |
| --- | --- | --- | --- | --- | --- |
| Primary-site-related factor |  |  |  | |  |
| Slow growth^1^ |  |  | 0 | |  |
| Moderate growth^2^ |  |  | 2 | |  |
| Rapid growth^3^ |  |  | 3 | |  |
| Laboratory data |  |  |  | |  |
| Normal |  |  | 0 | |  |
| Abnormal^4^ |  |  | 1 | |  |
| Critical^5^ |  |  | 2 | |  |
| Visceral metastases |  |  |  | |  |
| No |  |  | 0 | |  |
| Nodular |  |  | 1 | |  |
| Disseminated |  |  | 2 | |  |
| ECOG PS |  |  |  | |  |
| 0–2 |  |  | 0 | |  |
| 3–4 |  |  | 1 | |  |
| Previous chemotherapy |  |  |  | |  |
| No |  |  | 0 | |  |
| Yes |  |  | 1 | |  |
| Multiple skeletal metastases |  |  |  | |  |
| No |  |  | 0 | |  |
| Yes |  |  | 1 | |  |
|  |  |  |  |  |  |
| Total |  |  | 10 | |  |
| Risk Classification |  |  |  |  |  |
| Low-risk group |  |  | 0-3 | |  |
| Intermediate-risk group |  |  | 4-6 | |  |
| High-risk group |  |  | 7-10 | |  |
| ECOG = Eastern Cooperative Oncology Group, PS = performance status ^1^Hormone-dependent breast and prostate cancer, thyroid cancer, multiple myeloma, and malignant lymphoma. ^2^Lung cancer treated with molecularly targeted drugs, hormone-independent breast and prostate cancer, renal cell carcinoma, endometrial and ovarian cancer, sarcoma, and others. ^3^Lung cancer without treatment with molecularly targeted drugs, colorectal cancer, gastric cancer, pancreatic cancer, head and neck cancer, esophageal cancer, other urological cancers, melanoma, hepatocellular carcinoma, gall bladder cancer, cervical cancer, and cancers of unknown origin. ^4^C-reactive protein ≥0.4 mg/dL, lactate dehydrogenase ≥250 IU/L, or serum albumin <3.7 g/dL. ^5^Platelet count <100,000/µL, serum calcium level ≥10.3 mg/dL, or total bilirubin ≥1.4 mg/dL. | | | | |  |
|  |  |  |  |  |  |
|  |  |  |  |  |  |
|  |  |  |  |  |  |
|  |  |  |  |  |  |
|  |  |  |  |  |  |
|  |  |  |  |  |  |
|  |  |  |  |  |  |
|  |  |  |  |  |  |
