# Supplementary material for: Prediction of post-radiotherapy survival for bone metastases: a comparison of the 3-variable number of risk factors model with the new Katagiri scoring system
Source: J Radiat Res. 2021 Dec 31;63(2):303–11. doi: 10.1093/jrr/rrab121 (PMC8944300; doi:10.1093/jrr/rrab121)
Supplement: Supplementary_Table_2_rrab121 [file supplementary_table_2_rrab121.docx]

| Supplementary Table 2. Excluded patient characteristics (n = 109) | | | | | | |  |
| --- | --- | --- | --- | --- | --- | --- | --- |
|  |  |  |  |  | value | (% or range) |  |
| Age (years) | | |  |  | 65 | (12 - 92) |  |
| Sex, n (%) | | |  |  |  |  |  |
| Male | | |  |  | 60 | (55.0) |  |
| Female | | |  |  | 49 | (45.0) |  |
| Follow-up period (months) | | |  |  | 7.8 | (0 - 115.2) |  |
| Primary site, n (%) | | |  |  |  |  |  |
| Lung | | |  |  | 12 | (11.0) |  |
| Liver | | |  |  | 10 | (9.2) |  |
| Gastrointestinal | | |  |  | 15 | (13.8) |  |
| Prostate | | |  |  | 13 | (11.9) |  |
| Breast | | |  |  | 13 | (11.9) |  |
| Others | | |  |  | 46 | (42.2) |  |
| Visceral metastases, n (%) | | |  |  |  |  |  |
| No | | |  |  | 34 | (31.2) |  |
| Nodular metastasis | | |  |  | 48 | (44.0) |  |
| Disseminated metastasis | | |  |  | 27 | (24.8) |  |
| PS, n (%) | | |  |  |  |  |  |
|  | ECOG | 0 | KPS | 100-90 | 19 | (17.4) |  |
|  | ECOG | 1 | KPS | 80-70 | 37 | (33.9) |  |
|  | ECOG | 2 | KPS | 60-50 | 33 | (30.3) |  |
|  | ECOG | 3 | KPS | 40-30 | 18 | (16.5) |  |
|  | ECOG | 4 | KPS | 20-10 | 2 | (1.8) |  |
| Previous chemotherapy, n (%) | | |  |  |  |  |  |
| No | | |  |  | 21 | (19.3) |  |
| Yes | | |  |  | 88 | (80.7) |  |
| Multiple bone metastases, n (%) | | |  |  |  |  |  |
| No | | |  |  | 45 | (41.3) |  |
| Yes | | |  |  | 64 | (58.7) |  |
|  |  |  |  |  |  |  |  |
| The NRF model, n (%) | | |  |  |  |  |  |
| Group Ⅰ | | |  |  | 29 | (26.6) |  |
| Group Ⅱ | | |  |  | 42 | (38.5) |  |
| Group Ⅲ | | |  |  | 38 | (34.9) |  |
| Values are presented as median (range) unless otherwise noted. PS = performance status, ECOG = Eastern Cooperative Oncology Group, KPS = Karnofsky performance score, NRF = number of risk factors | | | | | | |  |
|  |  |  |  |  |  |  |  |
|  |  |  |  |  |  |  |  |
