# Supplementary material for: Prediction of post-radiotherapy survival for bone metastases: a comparison of the 3-variable number of risk factors model with the new Katagiri scoring system
Source: J Radiat Res. 2021 Dec 31;63(2):303–11. doi: 10.1093/jrr/rrab121 (PMC8944300; doi:10.1093/jrr/rrab121)
Supplement: Supplementary_rrab121 [file supplementary_rrab121.docx]

**Supplementary Data Captions**

**Supplementary Table 1.** **The new Katagiri scoring system**

**Supplementary Table 2. Excluded patient characteristics (n = 109)**

**Supplementary Figure 1. Kaplan-Meier curves of overall survival in all patients**

**Supplementary Figure 2. Kaplan-Meier curves of overall survival in excluded group**

NRF = number of risk factors. *Significant difference per the log-rank trend test (P < 0.05)
